# Stable Regulation of Cell Cycle Events in Mycobacteria: Insights From Inherently Heterogeneous Bacterial Populations

**DOI:** 10.3389/fmicb.2018.00514

**Published:** 2018-03-21

**Authors:** Michelle M. Logsdon, Bree B. Aldridge

**Affiliations:** ^1^Department of Molecular Biology and Microbiology, Tufts University School of Medicine, Boston, MA, United States; ^2^Department of Molecular Microbiology, Sackler School of Graduate Biomedical Sciences, Tufts University, Boston, MA, United States; ^3^Department of Biomedical Engineering, Tufts University School of Engineering, Medford, MA, United States

**Keywords:** cell size, mycobacteria, *Mycobacterium smegmatis*, BCG, chromosome organization, cell cycle, asymmetry, cell size control

## Abstract

Model bacteria, such as *E. coli* and *B. subtilis*, tightly regulate cell cycle progression to achieve consistent cell size distributions and replication dynamics. Many of the hallmark features of these model bacteria, including lateral cell wall elongation and symmetric growth and division, do not occur in mycobacteria. Instead, mycobacterial growth is characterized by asymmetric polar growth and division. This innate asymmetry creates unequal birth sizes and growth rates for daughter cells with each division, generating a phenotypically heterogeneous population. Although the asymmetric growth patterns of mycobacteria lead to a larger variation in birth size than typically seen in model bacterial populations, the cell size distribution is stable over time. Here, we review the cellular mechanisms of growth, division, and cell cycle progression in mycobacteria in the face of asymmetry and inherent heterogeneity. These processes coalesce to control cell size. Although *Mycobacterium smegmatis* and *Mycobacterium bovis* Bacillus Calmette-Guérin (BCG) utilize a novel model of cell size control, they are similar to previously studied bacteria in that initiation of DNA replication is a key checkpoint for cell division. We compare the regulation of DNA replication initiation and strategies used for cell size homeostasis in mycobacteria and model bacteria. Finally, we review the importance of cellular organization and chromosome segregation relating to the physiology of mycobacteria and consider how new frameworks could be applied across the wide spectrum of bacterial diversity.

## Introduction

Mycobacteria are unusual compared to other well-studied bacteria. They grow slowly, are not stained by dyes traditionally used to identify and differentiate bacteria, and have remarkably thick and waxy cell walls (Jankute et al., 2015). Although they have a similar rod shaped morphology as many model bacterial species, recent attention to their basic physiology demonstrates distinct growth modes, subcellular organization, cell cycle timing, division patterns, and size control. Due to these differences, much of the knowledge of basic cell cycle and growth processes developed in well-studied bacteria cannot be directly translated to mycobacteria. These models, however, provide valuable context for studying how the basic physiology of mycobacteria fits into the spectrum of previously studied microorganisms.

Mycobacterial infection remains a major threat to global health. Tuberculosis, caused by infection with *Mycobacterium tuberculosis*, is currently responsible for more deaths than any other single infectious agent (WHO, 2017). Key to mycobacterial virulence and drug tolerance is the ability to tightly control growth. Despite the importance of *M. tuberculosis* growth patterns contributing to infection dynamics, the basic lifecycle properties of mycobacteria are just beginning to be characterized. Until recently, mycobacteria were considered too slow growing and difficult to study to serve as an informative subject for basic cell physiology investigations. Division times of mycobacterial species vary wildly, and all are much longer than the model bacterium *E. coli*, who doubles every 20 min in rich medium. On the extreme end, *Mycobacterium leprae*, the causative agent of leprosy, doubles only once every 14 days and cannot be cultured *in vitro. M. tuberculosis* and Bacillus Calmette-Guérin (BCG) are slow growing, culturable mycobacteria with division times of 18–24 h in rich medium. *M. tuberculosis* is an airborne infectious organism that requires a high level of containment during experiments and can only be manipulated within a biosafety level 3 facility (BL3). BCG is often used as a proxy for *M. tuberculosis* in experiments because it is a closely related slow growing mycobacterium but is not pathogenic and does not require a specialized BL3 facility for experiments. However, BCG exposure can cause false positive reactions to the PPD skin test used to monitor exposure to tuberculosis (known as seroconversion, also seen in patients who have received the BCG vaccination) (Cohn, 2001). Therefore BCG can only be manipulated within a biosafety cabinet. *Mycobacterium smegmatis*, a non-pathogenic soil bacterium with a division time of about 3 h, is a commonly used model mycobacteria because it does not cause infection or seroconversion, can be used on a standard BL2 bench top, and allows experiments to be performed on a shorter, more manageable time scale. Much of the growth and division machinery is conserved between *M. smegmatis* and *M. tuberculosis* and the use of *M. smegmatis* as a model organism has allowed the field to progress rapidly in our understanding of the distinct mechanisms of growth and division in mycobacteria (Hett and Rubin, 2008). Additionally, the availability of microfluidic technologies has made basic cell biology studies more accessible, and in the past decade it has become apparent that growth variation within isogenic populations is an intrinsic property of mycobacteria (Aldridge et al., 2012; Kieser and Rubin, 2014). To gain a better understanding of their lifecycle and persistence, it is imperative that we approach mycobacteria as unique and complex organisms.

**FIGURE 1:**
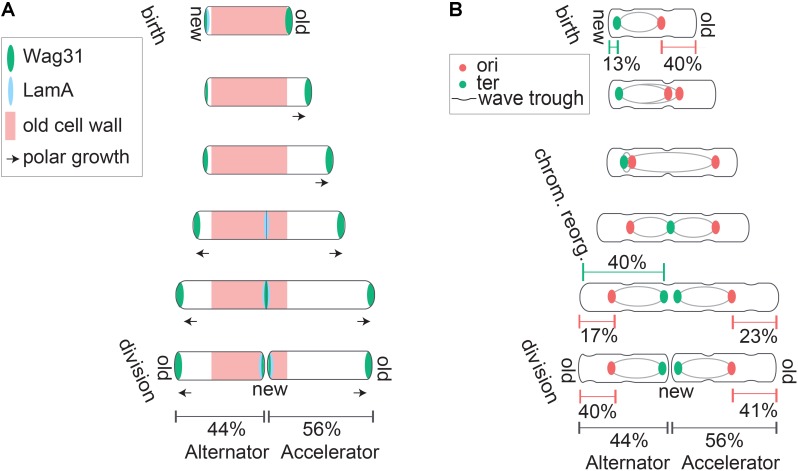
Asymmetric polar growth and division in mycobacteria. **(A)** Mycobacteria grow primarily from subpolar regions (marked with arrows), and elongate preferentially from the old pole. Asymmetric polar growth is directed by elongation and divisome components Wag31 (green) and LamA (blue). At birth, LamA actively inhibits growth from the new pole while Wag31 localizes to the old pole and organizes the elongation complex to promote growth at the old pole. Later in the cell cycle LamA inhibition of new pole growth is relieved, while Wag31 accumulates and promotes growth from the new pole. Finally, LamA and Wag31 accumulate at the septum region where Wag31 assembles the elongation complex while LamA inhibits growth from this site that becomes the new pole after division. **(B)** Mycobacteria divide asymmetrically and organize their chromosomes asymmetrically within the cell. Asymmetric division is partially organized by surface wave troughs inherited from previous generations. Cells divide at the centermost wave trough along the cell body. At birth, the ori region of the chromosome (red) is positioned midcell, but closer to the old pole, and the ter (green) is positioned near the new pole. The chromosome reorganizes part way through the cell cycle. Ori’s partition and travel to asymmetric quarter cell positions and the terminus translocates to an asymmetric midcell location that corresponds with surface wave trough and septum placement. At division, proportional chromosome localization is reestablished in daughter cells despite the differential sizes of accelerator and alternator sisters.

Differences in physiology between mycobacteria and model bacteria include mechanisms of cell division and growth. A key characteristic of mycobacterial physiology is their striking pattern of asymmetric growth and division (Aldridge et al., 2012; Kieser and Rubin, 2014; Meniche et al., 2014; Manina et al., 2015; Rego et al., 2017). Mycobacteria elongate asymmetrically, preferentially from the old pole (Aldridge et al., 2012; Meniche et al., 2014; Botella et al., 2017; Rego et al., 2017). The new pole experiences a lag in growth before initiating growth partway through the cell cycle (**Figure 1A**) (Aldridge et al., 2012; Botella et al., 2017). The mechanisms controlling initiation or “licensing” of new pole growth are not well understood. In *M. smegmatis* the new pole grows at a slower rate from licensing to division than the old pole, while in *M. tuberculosis*, the rate of new pole growth catches up to the rate of old pole growth preceding division (Botella et al., 2017). Due to differential growth characteristics in cell poles preceding division, sister cells inheriting the mother’s old pole have different polar growth characteristics than sister cells inheriting the new pole (Aldridge et al., 2012; Kieser and Rubin, 2014). The “accelerator” sister cell inherits the mother’s old pole and is larger and faster growing while the “alternator” sister inherits the mother’s new pole and is smaller and slower growing (**Figure 1A**) (Aldridge et al., 2012). This review will address our current understanding of mycobacterial cell biology regarding the generation of asymmetry among closely related cells, impacts of asymmetry on population structure, coordination of innate asymmetry and key cell cycle events, and regulation of cell size in the face of differential growth and size characteristics in mycobacteria.

## The Basis of Asymmetry

The molecular mechanisms underlying asymmetric growth and division in mycobacteria are just beginning to be elucidated. Many growth and division factors localize in specific patterns to facilitate temporal regulation of growth from each pole. Most well studied bacteria, including *E. coli*, *B. subtilis*, and *C. crescentus* elongate laterally along the length of the cell wall using actin like protein MreB (Daniel and Errington, 2003; Takacs et al., 2010; Dominguez-Escobar et al., 2011; Garner et al., 2011; Wang et al., 2012; White and Gober, 2012; Kysela et al., 2013; Errington, 2015). Mycobacteria, on the other hand, elongate primarily from subpolar regions adjacent to cell poles where the coiled-coil protein Wag31 (also called DivIVA) serves as a scaffold for the elongation complex (Kang et al., 2008; Meniche et al., 2014). Wag31 is targeted to the cell pole through recognition of membrane curvature, where it anchors peptidoglycan, arabinogalactan, and mycolic acid synthesizing enzymes (MurG, GlfT2, and Pks13, respectively) (Meniche et al., 2014). Wag31 preferentially localizes to the old cell pole, consistent with the observation that the old pole serves as the primary site of cell elongation throughout the mycobacterial cell cycle (**Figure 1A**) (Kang et al., 2008; Meniche et al., 2014). Wag31 moves to the new pole at septation to prepare for eventual new pole elongation (**Figure 1A**) (Kang et al., 2008; Santi et al., 2013). Several of the proteins anchored by Wag31, including arabinoglactan synthesizing protein GlfT2, specifically associate with a specialized membrane domain called the pure membrane free of cell wall components (PMf) (Hayashi et al., 2016). GlfT2 is localized to growing old poles, indicating that lipid biosynthetic reactions required for cell envelope synthesis are targeted to regions of active cell growth (Hayashi et al., 2016). Wag31 interacts with the cell wall associated membrane fraction and not the PMf (Hayashi et al., 2016). It has yet to be determined how the distinct PMf and cell wall associated membrane fractions work together to promote cell growth. Regardless, it is clear that the localization of a metabolically active membrane domain, as well as scaffold protein Wag31, provides a means of targeting cell growth preferentially to cell poles.

Many mycobacterial growth studies employ fluorescently tagged Wag31 as an elongation and early cell division marker because of its important role in organizing cell elongation complexes (Santi et al., 2013; Meniche et al., 2014; Santi and McKinney, 2015; Botella et al., 2017). Wag31-fluorescent protein fusions form clear and bright fluorescent bands at sites of cell growth and septation. However, the function of Wag31 is very sensitive to fluorescent protein fusion and overexpression. Adding a GFP tag to the C-terminus of native Wag31 leads to aberrant localization of the Wag31-GFP band at the new pole, causing the new pole to become the primary site of growth (Meniche et al., 2014). The C-terminal tagged Wag31-GFP also caused a 40% decrease in cell elongation rate, increased cell width, and an abnormal curved shape in cells (Meniche et al., 2014). However, a merodiploid strain with an unaltered native copy of Wag31 and a fluorescent protein tagged copy of Wag31 integrated elsewhere on the chromosome localized as expected to the old pole and restored the wild type cell shape phenotype (Meniche et al., 2014).

A recent study used super-resolution microscopy and fluorescent D-amino acids (FDAAs) to stain a *M. smegmatis* reporter strain expressing an episomal mCherry tagged Wag31 (Botella et al., 2017). FDAAs are a recently developed class of reporters that are incorporated directly into growing peptidoglycan to identify specific regions of elongation along a bacterial cell (Siegrist et al., 2013; Kuru et al., 2015; Botella et al., 2017). In mycobacteria, FDAA staining was used to differentiate growth from old versus new poles. Cells expressing a fluorescently tagged Wag31 elongated more symmetrically between old and new poles compared to wild type *M. smegmatis*, which elongated preferentially from the old pole (Botella et al., 2017). Together, these bodies of work suggest that Wag31 localization and function is particularly sensitive to fluorescent protein tags, because several of the constructs utilized significantly alter cell growth and polarity (Santi et al., 2013; Meniche et al., 2014; Santi and McKinney, 2015; Botella et al., 2017). Adding a fluorescent protein to any important enzyme can alter enzyme activity, localization, and cell physiology (Landgraf et al., 2012), and this seems especially true of Wag31. A loss of cellular growth asymmetry could have major consequences for bacterial population structure and fitness; therefore we must be cautious in interpreting results from studies performed with fluorescently tagged Wag31 in mycobacteria.

Asymmetric polar growth in mycobacteria is created by a protein conserved among mycobacteria but with no homologs in other characterized bacteria. This protein, called LamA (named for Loss of Asymmetry Mutant A), is a member of the mycobacterial division complex where it actively inhibits growth at the new pole (**Figure 1A**) (Rego et al., 2017). LamA interacts with penicillin binding protein PonA1. Deletion of LamA allows Wag31 to be recruited to the new pole more rapidly, indicating that LamA works to delay assembly of the elongation complex at the new pole (Rego et al., 2017). LamA inhibition of new pole growth in WT cells allows the old pole to grow preferentially, creating the characteristic asymmetric polar growth patterns of mycobacteria (**Figure 1A**).

Though identifying functions of proteins Wag31 and LamA provides us with a glimpse of the networks involved in regulating asymmetric growth in mycobacteria, the extent of their interactions with elongation complexes have yet to be fully elucidated. A current understanding of the processes involved in cell elongation complexes is described in an excellent review by Kieser and Rubin (2014). The enzymatic activity of a large number of proteins is required for synthesizing peptidoglycan, arabinogalactan, and mycolic acid layers in mycobacteria. Asymmetry-promoting proteins Wag31 and LamA likely interact with enzyme complexes and membrane domains involved in cell wall and cell membrane synthesis (Meniche et al., 2014; Hayashi et al., 2016). It will be interesting to understand exactly how previously identified cell elongation networks are asymmetrically regulated at each pole and how growth cues such as nutrient status may interface with these pathways.

In addition to growing asymmetrically, mycobacteria also divide asymmetrically and undergo a fast, mechanical v-snapping process of daughter cell separation (Aldridge et al., 2012; Richardson et al., 2016; Zhou et al., 2016). There is evidence to suggest that sites of asymmetric division are not just established at division, but are inherited from previous generations. Atomic force microscopy (AFM) of the mycobacterial cell surface revealed that cell surface wave troughs can determine division site selection (Eskandarian et al., 2017). Repeating surface waveform troughs are inherited over several generations and the total number of troughs scales with cell size (**Figure 1B**) (Eskandarian et al., 2017). Cells divide at the centermost trough and the other troughs are passed down to subsequent generations to be used as a future division site (**Figure 1B**) (Eskandarian et al., 2017). Furthermore, while cell wall synthetic enzymes GlfT2, MurG, and Pks13 show primary localization to subpolar regions where growth occurs, re-examination of this data indicates secondary localization peaks at quarter cell positions (Meniche et al., 2014). It has yet to be investigated if these elongation complex positions co-localize with surface wave troughs, as a potential means of using division troughs to position cell elongation complexes a generation in advance.

Wild type *M. smegmatis* cells divide at the central surface wave trough while cells deficient in chromosome partitioning can divide at off-center wave troughs, indicating a regulatory role for chromosome segregation in division site selection (Eskandarian et al., 2017). Mycobacteria lack homologues of defined nucleoid occlusion systems (Noc proteins) and minicell (Min) proteins used by other bacteria to prevent chromosome splicing during cell division (Wu and Errington, 2011; Monahan et al., 2014; Schumacher, 2017). Despite the lack of known nucleoid occlusion proteins, *M. smegmatis* cells exhibit a clear relationship between asymmetric chromosome positioning and asymmetric division placement (Eskandarian et al., 2017; Logsdon et al., 2017) (**Figure 1B**). Division occurs in the wave trough nearest the local DNA minimum even in mycobacteria deficient in chromosome segregation, demonstrating the existence of a mechanism to prevent damage to the chromosome during cell division (Eskandarian et al., 2017).

Together, these studies support the idea that chromosome dynamics contributes to asymmetric cell division in mycobacteria. The chromosome is a large, essential macromolecule that may play a key role in determining the layout of many aspects of the intracellular space (Campos and Jacobs-Wagner, 2013). Different bacterial species exhibit a variety of chromosome orientations and arrangements, reflecting the diversity of bacterial cell shapes, sizes, and growth modes. For example, in *E. coli* replication is initiated midcell and continues with two replication forks moving independently before terminating midcell (Reyes-Lamothe et al., 2008). This pattern represents an L-ori-R (left-origin-right) orientation. In *C. crescentus*, DNA replication begins near the flagellar old pole during the swarmer to stalked cell transition. The replisome migrates midcell while the newly replicated ori “flips” and travels across the cell from the stalked pole to the newly formed flagellar pole (Bowman et al., 2008). The *C. crescentu*s chromosome organization pattern represents an ori-ter-ter-ori (origin-terminus-terminus-origin) orientation. Other bacteria with ori-ter-ter-ori chromosome organizations include *Pseudomonas aeruginosa*, chromosome 1 of *Vibrio cholerae*, and *Myxococcus xanthus* (Fogel and Waldor, 2005; Harms et al., 2013; Vallet-Gely and Boccard, 2013). *B. subtilis*, on the other hand, oscillates between an ori-ter-ter-ori and L-ori-R pattern, depending on cell cycle stage (Wang et al., 2014).

Chromosome organization and partitioning likely direct many cellular processes. These processes include ensuring genetic inheritance, directing cell division, regulating transcription, and targeting transcripts and their encoded proteins to their necessary location within the cell (Montero Llopis et al., 2010; Nevo-Dinur et al., 2011; Govindarajan et al., 2012; Campos and Jacobs-Wagner, 2013). For example, the order of genes along the chromosome is highly conserved in gammaproteobacteria and correlated with the temporal regulation of gene expression (Sobetzko et al., 2012). The most highly conserved aspect of global gene arrangements is distance from either the ori or ter region (Sobetzko et al., 2012). Proximity of a gene to the ori or ter region on the chromosome could contribute to temporal regulation of its expression through gene dosage, which refers to the difference in copy number of genes located near the ori versus the ter while DNA replication is ongoing (Slager and Veening, 2016). Genes in the ori region are replicated first and thus periodically have two copies while genes near the ter only have one copy until the end of replication. In *Vibrio cholerae*, the ori proximal location and expression of ribosome genes is essential for proper growth and host invasion. Moving half of the genes required for ribosome proteins to distal sites along the chromosome leads to significant defects in growth and invasion processes (Soler-Bistue et al., 2015). In mycobacteria, a notable operon positioned near the ori is called the division cell wall (dcw) cluster and contains many genes whose products are required for cell wall synthesis and division, including *ftsZ, ftsQ, ftsW, murC, murG, murD, murX, murF, murE*, and *wag31* (Kang et al., 2008). The functional implications of maintaining this growth and division operon near the ori have yet to be investigated in mycobacteria, but it stands to reason that this position could allow regulation of expression through gene dosing and/or proper localization of important growth and division proteins.

The molecular machinery responsible for chromosome partitioning and placement within most studied bacterial cells (with the notable exception of *E. coli*) is the ParABs system (Lee and Grossman, 2006; Mierzejewska and Jagura-Burdzy, 2012; Iniesta, 2014; Lagage et al., 2016). The ParABs system in mycobacteria consists of two 16 bp centromere-like *parS* sequences located near the ori on the chromosome (Casart et al., 2008). The ParB DNA binding protein specifically recognizes and binds the *parS* sequences, and its activity is negatively regulated through Ser/Thr kinase phosphorylation (Baronian et al., 2015). ParA, a Walker ATPase, interacts with ParB and directs segrosome partitioning (Ginda et al., 2017). The movement of the ori across the cell to the new pole allows the cell to divide into daughter cells with two neatly packaged chromosomes. ParB colocalizes with the nucleoid associated protein responsible for maintaining chromosome compaction, HupB, indicating a role for HupB in ori segregation (Holowka et al., 2017). The ParABs system is also strongly integrated with aspects of the elongation and division machinery in *M. smegmatis*. Knocking out or overproducing ParA impairs cell division, resulting in filamentous and multinucleoidal cells that have failed to divide (Maloney et al., 2009). ParA interacts directly with the polar protein responsible for the organization of growth and division factories, Wag31 (Ginda et al., 2013). Therefore, studying chromosome partitioning could give us new insight into the integration of chromosome organization and cell division processes.

Several groups have characterized the chromosome organization of mycobacteria using live cell microscopy, and multiple models of chromosome subcellular organization have been proposed. Santi and McKinney (2015) used a fluorescent protein tagged ParB/Wag31 dual reporter strain to conclude that the ori is localized midcell before replication and after replication, both ori’s segregate to mirror symmetric quarter cell positions to prepare for midcell localization in the two daughter cells. Chromosomal locus attB1, located at 245° on the left lobe, was found initially localized near the new pole before translocating midcell during replication. Based on the midcell position of the ori and new pole position of the left lobe, they concluded the *M. smegmatis* chromosome organization had a R-ori-L configuration, as in *E. coli*. However, the use of a fluorescent protein labeled Wag31 may have affected the growth polarity in these reporter cells (Meniche et al., 2014; Botella et al., 2017).

Recent studies using ParB (Trojanowski et al., 2015; Ginda et al., 2017) or fluorescent reporter operator system (FROS)-ori (Logsdon et al., 2017) reporters independently converged on a model in which the ori is positioned asymmetrically and closer to the new pole at birth (**Figure 1B**). A few differences between the reporters utilized and results generated should be noted. The ParB reporter consistently shows ori splitting and segregation much earlier than the FROS ori reporter (Santi and McKinney, 2015; Trojanowski et al., 2015, 2017; Ginda et al., 2017; Logsdon et al., 2017). Many cells inherited two ParB foci, indicating these origins had already undergone a second round of replication and a second segregation event before division, which was not observed using the FROS-ori reporter (Ginda et al., 2017; Logsdon et al., 2017; Trojanowski et al., 2017). In these cases, the ParB reporter displayed differing frequencies of re-splitting before division between studies ranging from 0% (Trojanowski et al., 2015), to 11% (Trojanowski et al., 2017), to 75% (Ginda et al., 2017). It is not apparent why these reporters produce different segregation dynamics, however, the asymmetric ori positioning was consistent across studies (Trojanowski et al., 2015, 2017; Ginda et al., 2017; Logsdon et al., 2017). Identification of ori positioning, along with the use of a FROS-terminus reporter, allowed for a more comprehensive investigation of subcellular chromosome localization and strongly supports an ori-ter-ter-ori chromosome orientation (Logsdon et al., 2017). Localization data show the terminus positioned near the new pole at birth followed by a rapid translocation event to the midcell partway through the cell cycle (**Figure 1B**). This asymmetric ori-ter-ter-ori chromosome positioning is not unique to mycobacteria, as it has been previously described in *Myxococcus xanthus* (Harms et al., 2013).

How do chromosome organization patterns in mycobacteria contribute to cell division? The observed ori-ter-ter-ori orientation pattern is asymmetric within the cell throughout the entire cell cycle (Ginda et al., 2017; Logsdon et al., 2017). Though the chromosome terminus translocates midcell, it is not located precisely halfway between each pole. Rather, it is slightly shifted toward the new pole, in a manner that corresponds with asymmetric septum positioning at cell division (**Figure 1B**) (Logsdon et al., 2017). In many cells, the terminus shifts midcell but remains as a single focus (even after replication has completed) until 15–30 min before cell division. Just before division, the two terminus foci move slightly apart and the septum forms directly between them.

Terminus translocation midcell could be a very early determinant of the division site. How the terminus finds this location is unclear – there are systems identified for origin localization in mycobacteria and other bacteria but not terminus localization. The terminus and inherited wave troughs may interact to target termini to the future division spot. Additionally, interactions of the terminus with members of the divisome to prevent division over chromosomes have not been characterized. Mycobacteria do not have homologs of any identified nucleoid occlusion systems but it stands to reason that they have an alternate system that only allows septum formation in the DNA deficient space between termini at division. Overall, many studies indicate that asymmetry is an integral part of mycobacterial physiology that sets this genus apart from other well studied, symmetric bacteria.

## Behavioral Consequences of Asymmetry

The establishment of systematic asymmetry in mycobacterial cells likely has functional consequences on behaviors of both single cells and populations of mycobacteria. One consequence of cellular asymmetry is the creation of cellular heterogeneity, even between presumably isogenic sister cells. Phenotypic heterogeneity creates subpopulations of mycobacteria from a single progenitor with varying size, growth, and cell cycle properties (Aldridge et al., 2012; Richardson et al., 2016). Deterministic generation of heterogeneity among populations of cells may serve as a bacterial “bet hedging” mechanism, allowing a subpopulation of cells with a particular phenotype to survive environmental stress (Kysela et al., 2013; Richardson et al., 2016). Differentiation of cell phenotypes leading to bet hedging is apparent in the model organism *C. crescentus*. Upon division, two distinct daughter cell types are generated. One daughter cell is immobile and has a polar stalk while the other has a polar flagellum used for swarming motility. The stalked daughter remains attached to its current environment, while the swarmer daughter takes a risk to swim away and colonize a new, potentially preferential environment (Kysela et al., 2013). In this way, if environmental conditions wipe out all cells at the initial site of colonization, half the cells are already searching for a viable site where they can grow and replicate.

The functional consequences of asymmetric growth and division in mycobacteria are perhaps more subtle. Differences in cell size and cell cycle stage at the time of antibiotic treatment contribute to differential antibiotic susceptibility (Aldridge et al., 2012; Richardson et al., 2016). When treated with cell wall acting antibiotics, faster growing accelerator cells are more sensitive to cycloserine and meropenem than their sister alternator cells (Aldridge et al., 2012). Additionally, *M. smegmatis* cells exhibit differential susceptibility to rifampicin treatment. Rifampicin tolerant cells are larger at birth and inherit older growth poles compared to susceptible cells (Richardson et al., 2016). Together, these studies indicate that asymmetric growth and division within a population of mycobacteria allows certain cells to survive an antibiotic stressor while others with different phenotypic characteristics perish.

The role of asymmetric growth and division in *M. tuberculosis* infection is just beginning to be investigated. Recently, Vijay et al. (2017) examined cell size distributions of 158 clinically isolated *M. tuberculosis* strains and observed that *M. tuberculosis* cell sizes are smaller and less variable after growing in rich liquid broth compared to their sizes directly from patient sputum or passaged through J774 mouse macrophages. These measurements suggest that clinical, non-laboratory adapted strains of *M. tuberculosis* may increase phenotypic heterogeneity in response to host stress conditions. Additionally, cell length and heterogeneity increased in clinically isolated multidrug resistant *M. tuberculosis* (MDR-TB) strains when exposed to the first line antibiotics rifampicin and isoniazid (Vijay et al., 2017). Cell size variability further increased in MDR-TB subjected to both macrophage infection and rifampicin treatment (Vijay et al., 2017). This increase in *M. tuberculosis* size and variability suggests a synergistic effect between drug stress and infection. Thus, phenotypic heterogeneity manifested in cell size in addition to defined genetic mutations, may shape *M. tuberculosis* drug and host tolerance during infection.

Manina et al. (2015) also investigated the effect of host and drug stress on *M. tuberculosis* phenotypic heterogeneity in defined conditions with the lab adapted Erdman strain of *M. tuberculosis*. A GFP tagged rRNA reporter was used to demonstrate increases in *M. tuberculosis* cell-to-cell heterogeneity in nutrient limited medium, stationary phase culture, macrophage infection, and mouse lung infection compared to growth in rich medium (Manina et al., 2015). Increased *M. tuberculosis* cellular variability may be a direct response to stressful environmental conditions, although the physiological and molecular response leading to phenotypic changes in *M. tuberculosis* are poorly understood. Increased phenotypic heterogeneity observed under host and drug stress could be mediated through subtle changes in patterns of *M. tuberculosis* asymmetric growth and division. These studies suggest that amplifying phenotypic heterogeneity may allow a subpopulation of *M. tuberculosis* cells to persist within a host during infection and drug treatment. If true, it is tempting to speculate that identifying and targeting particularly tolerant cell subpopulation could clear TB infection more efficiently.

Finally, molecular support for the hypothesis that phenotypic heterogeneity promotes bacterial survival comes from studies using the symmetrically growing LamA mutant. Δ*lamA M. smegmatis* and *M. tuberculosis* show less size heterogeneity than wild type cells and are killed more rapidly and uniformly by rifampicin and cell wall acting antibiotics (Rego et al., 2017). This molecular study, together with population level studies, support the hypothesis that asymmetric polar growth actively creates heterogeneity amongst bacterial cells to serve as a bulk survival mechanism, ensuring a subpopulation of *M. tuberculosis* can persist in stressful environments.

## Cell Cycle

**FIGURE 2:**
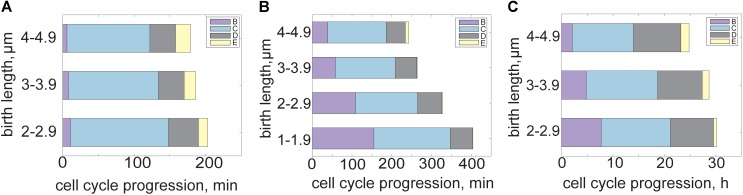
Mycobacterial cell cycle and growth dynamics shift in fast versus slow growth conditions. **(A)** Bar plot of *M. smegmatis* cell cycle progression in rich medium. B period (pre-replication, purple) is short while C (active DNA replication, blue) encompasses most of the cell cycle. D period (post replication, gray) is relatively constant across cell birth sizes but E period (re-initiation, yellow) is associated with cells born larger. **(B)** Bar plot showing *M. smegmatis* cell cycle progression and variability across cell sizes in carbon limited medium. B period is disproportionally extended in carbon limited growth conditions, as smaller cells require more time to initiate DNA replication. E period encompasses less of the cell cycle during carbon limitation. **(C)** Bar plot showing BCG cell cycle progression across cell sizes in rich medium. Duration of B period is associated with cell size, with cells born smaller waiting longer to initiate DNA replication. C and D periods are relatively constant in the BCG cell cycle and E period is infrequent. Data from Logsdon et al. (2017).

To coordinate chromosome duplication with changes in cell size, growth rate, and variability in stressful growth conditions, mycobacteria alter the dynamics of their cell cycle. These changes are essential to adapt to a wide variety of environments and ensure faithful replication and genetic inheritance. The bacterial cell cycle is usually described as three basic phases: B period occurs after division and before DNA replication, C is the period of active DNA replication, and D occurs after DNA replication but before cell division. In *M. smegmatis*, a fraction of cells begin a new round of DNA replication before division and their daughters subsequently skip B period and continue straight into C (Santi et al., 2013; Sukumar et al., 2014; Trojanowski et al., 2015; Richardson et al., 2016; Logsdon et al., 2017). We named this occurrence the E period (Sukumar et al., 2014). Overall, *M. smegmatis* spends the majority of the cell cycle in C period replicating the chromosome while B and D period are relatively short (**Figure 2A**) (Santi et al., 2013; Sukumar et al., 2014; Logsdon et al., 2017; Trojanowski et al., 2017). E period occurs more frequently and lasts longer in cells that were large at birth (**Figure 2A**) (Logsdon et al., 2017).

A similar but distinct overlap in replication cycles was recently reported in *M. smegmatis*. Trojanowski et al. (2017) observed multifork replication in 11% of *M. smegmatis* cells growing in rich medium. Multifork replication occurs when a second (or third or fourth) round of replication begins before the first has completed (Helmstetter, 1968; Trojanowski et al., 2017). Multifork replication is typically associated with fast growing bacterial species as an adaption to fast growth conditions, in which division time is shorter than the time required to replicate the chromosome. By initiating several rounds of replication before the first has completed, daughter cells inherit an already partially replicated chromosome, preventing the rate of division from outpacing the rate of chromosome duplication. The differing observations of E period and multifork replication in mycobacteria come from studies using different fluorescent DNA replication reporters and imaging/growth conditions, making it difficult to make direct comparisons (Santi et al., 2013; Sukumar et al., 2014; Logsdon et al., 2017; Trojanowski et al., 2017). Even so, both multifork replication and E period are associated with large cells, and the frequency of both re-initiation events decreases under nutrient limitation (Logsdon et al., 2017; Trojanowski et al., 2017).

It is uncommon to observe re-initiation (E period) or multifork replication in slow growing bacteria, particularly a species with a period of DNA replication (C period) shorter than the interdivision time. Mycobacteria therefore break the paradigm in bacterial cell physiology that supposes only fast growing organisms engage in multifork replication. The effects of gene dosage on transcription are multiplied during multifork replication (ori:ter ratios are 4:1 instead of 2:1 or 1:1) and could lead to an overabundance of transcripts from genes located near the ori and set into motion any number of cellular regulatory programs (Slager and Veening, 2016). Ultimately, the effect of gene dosage and transcript abundance could create an additional layer of heterogeneity in the subpopulation of mycobacterial cells experiencing reinitiation. Because *M. smegmatis* cells engaging in multifork replication comprise only a small percent of the total population (11%) many more cells need to be studied to determine the functional utility of this behavior (Trojanowski et al., 2017). Overall, multifork replication may serve a different purpose or evolutionary advantage in slow growing mycobacteria compared to fast growing model bacteria and these cell cycle patterns require further investigation.

Several other distinct cell cycle timing changes occur in mycobacteria under nutrient limitation. Studying cells grown under carbon limitation presents a chance to examine cell cycle dynamics in environmental conditions more similar to those encountered during early infection. When grown in carbon-limited medium, the average *M. smegmatis* interdivision time increases by 2 h (Logsdon et al., 2017). Rather than slowing all periods of the cell cycle equally, B period is extended disproportionally (**Figure 2B**). The proportion of the cell cycle spent in B period is negatively correlated with cell birth size, meaning that small cells require significantly more time before initiating DNA replication than cells born large (**Figure 2B**) (Logsdon et al., 2017). However, post initiation time (C+D+E periods) remains constant and independent of cell size at birth (**Figure 2B**). The extended delay before initiation could be an adaption strategy to early infection, during which there is evidence that mycobacteria slow or halt their growth to avoid host detection (Rohde et al., 2012).

The slow growing mycobacterial species BCG is often used as a non-pathogenic model for another slow growing pathogenic mycobacteria, *M. tuberculosis*. The BCG interdivision time is longer than *M. smegmatis* (and comparable to that of *M. tuberculosis*), spanning from 15 to 20 h with a C period lasting an average of 9.4 h (**Figure 2C**) (Nair et al., 2009; Logsdon et al., 2017). The B period shows an extended duration and negative correlation with cell size at birth while the total time spent initiation to division remains constant across cell size, as in carbon limited *M. smegmatis* (**Figure 2C**) (Logsdon et al., 2017). The very long interdivision and DNA replication times in slow growing species present a long standing puzzle for mycobacteriologists (Ditse et al., 2017). A recent study of the *M. tuberculosis* DNA polymerase DnaE1 showed that the process of nucleotide incorporation is not the rate-limiting step in DNA replication or division times, because recombinant *M. tuberculosis* DnaE1 polymerase works faster *in vitro* than the *E. coli* DNA polymerase PolIIIα (Rock et al., 2015). Therefore there must be additional factors limiting the rate of DNA replication and cell cycle progression in slow growing mycobacteria that should be identified with future work. The necessity of coordinating DNA replication with chromosome partitioning, elongation, division, and metabolic status likely plays a role in determining rates of DNA replication in slow growing mycobacteria.

During growth in nutrient-limited environments, cell cycle timing and cell sizes become more heterogeneous. The mycobacterial cell cycle (particularly in slow growing mycobacteria) may be innately asynchrononus. The length of B period varies significantly based on cell birth size, and daughter birth sizes are intrinsically unequal, thus cell cycle timing is also intrinsically unequal (Aldridge et al., 2012; Logsdon et al., 2017). This asynchrony may provide an additional level of heterogeneity to promote population survival. The cell cycle stage at the start of rifampicin treatment is a strong predictor of bacterial survival on a single cell level (Richardson et al., 2016). It is therefore intriguing that slow growing mycobacteria BCG or carbon limited *M. smegmatis* have a means of generating cells in many stages of the cell cycle within a single colony. These studies suggest that mycobacterial cell cycle dynamics could determine, on a single cell level, which bacteria will survive nutrient or antibiotic pressure. Elucidating the effect of cell cycle dynamics on bacterial stress tolerance and survival will require additional single cell experiments and analysis (Taheri-Araghi et al., 2015b). With continued study focusing on the timing of cell cycle components in individual cells, we can begin to elucidate how mycobacteria regulate their lifecycle to persist in the face of environmental stress.

## Cell Size Control

It is clear that mycobacteria must manage variability in size, growth, and cell cycle timing in the wide range of conditions potentially encountered during infection. Coordination of these essential cell processes ensures reproducibility of cell sizes in the face of environmental stress. Even under rich growth conditions, asymmetric elongation and division in mycobacteria give rise to increased variability in cell size compared to model bacteria such as *E. coli*, *B. subtilis*, and *C. crescentus*, yet the distribution of this variability is stable over time (Aldridge et al., 2012; Amir, 2014; Campos et al., 2014; Taheri-Araghi et al., 2015a). Cell size homeostasis is an important aspect of the physiology of all living cells because without a mechanism of regulation, cell sizes and physiological properties would rapidly diverge and become unsustainable. Mycobacterial populations maintain stability in cell size variation despite larger sister cells elongating faster and smaller sister cells elongating slower, indicating a strong cellular mechanism of size regulation to prevent differences in size and growth rate from compounding and diverging over time. What mechanism is used by mycobacteria to maintain control of cell sizes when their variability in cell size increases under stress?

In well-studied, model bacterial species the distribution of cell birth sizes is quite narrow. *E. coli* birth size distribution has a coefficient of variation (CV) of 12% and *C. crescentus* maintains a CV of 14%, despite being another asymmetrically dividing bacterial species (Campos et al., 2014; Iyer-Biswas et al., 2014). This tightly controlled population structure has fascinated microbiologists for decades and lead to several attempts to model the factors controlling bacterial cell division and cell size (Schaechter et al., 1958; Koch and Schaechter, 1962; Cooper and Helmstetter, 1968; Donachie, 1968). However, it was not clear how cell size control models, which are well studied in other bacteria, would translate given the physical asymmetry of mycobacteria.

Mathematical models of bacterial cell size control have been introduced beginning in the 1960s (Koch and Schaechter, 1962; Cooper and Helmstetter, 1968; Donachie, 1968; Amir, 2014; Campos et al., 2014; Ho and Amir, 2015; Taheri-Araghi et al., 2015a; Harris and Theriot, 2016; Sauls et al., 2016; Soifer et al., 2016; Wallden et al., 2016; Zheng et al., 2016; Jun et al., 2018). To date, most models of bacterial cell size control have been built on the assumption that bacteria grow exponentially at a single-cell level (Schaechter et al., 1958). However, time resolution of imaging in *M. smegmatis* is limited due to phototoxic effects, such that single cell traces cannot distinguish between linear and exponential modes of growth. Though growth data on a single-cell level have been reported as either an averaged growth rate (length/time) or an exponential growth constant (1/time) depending on the analysis type, consensus whether mycobacterial growth per cell is linear or exponential is lacking. (Aldridge et al., 2012; Santi et al., 2013; Wakamoto et al., 2013; Logsdon et al., 2017; Priestman et al., 2017). There are other advanced techniques used to definitively determine the mode of mass increase within a bacterial cell over time (Godin et al., 2010; Cermak et al., 2016), however, these have not been applied to mycobacteria.

Recent studies have taken advantage of growth measurements from many cells to compare against expected relationships between generation time and cell size of exponentially or linearly growing bacteria. Studies using bulk measurements and single cell traces with model selection are in agreement with exponential growth expectations compared to a linear growth model (Logsdon et al., 2017; Priestman et al., 2017). Interestingly, both found that the fraction of *M. smegmatis* cells with the longest interdivision times deviate from exponential growth and appear more linear (Logsdon et al., 2017; Priestman et al., 2017). It would be interesting to determine if another cellular growth mechanism in these slowly dividing cells affects apparent linear versus exponential growth. Perhaps very large cells inherited old growth poles or experienced a lag in growth and could have distinct stress responses compared to their exponentially growing counterparts (Kysela et al., 2013).

**FIGURE 3:**
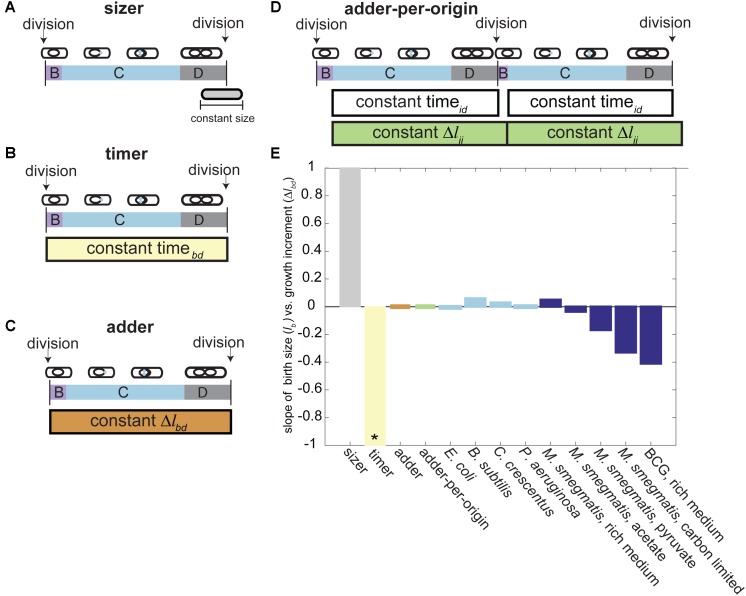
Models of bacterial cell size control. **(A)** In the sizer model, cells divide once they reach a constant size (e.g., cells divide once reaching 7 μm in length). **(B)** In the timer model, cells divide a constant time after birth (e.g., cells divide 100 min after birth). **(C)** In the adder model, cells divide after adding a constant growth increment from birth (e.g., cells divide after adding 3 μm to their birth length). **(D)** In the adder-per-origin model, cells add a constant increment from initiation to initiation and then divide a constant time after initiation (e.g., cells divide 100 min after initiation, and grow 3 μm between initiation events). **(E)** Bar plot depicting the slope of cell birth length (Δ*l*_b_) versus birth to division adder increment (Δ*l*_bd_) for several size control models and bacterial populations. Model predictions for the slope of Δ*l*_b_ vs. Δ*l*_bd_ are shown on the left of the graph. The sizer model (left, gray bar) predicts a slope of 1, because cells born smaller must grow more to reach the required size for division (Koch and Schaechter, 1962; Sauls et al., 2016). The timer model (yellow bar) predicts a slope of –1. In a population of cells where growth rate is exponentially correlated with cell size, larger cells are expected to grow more than smaller cells in the same amount of time, giving a negative slope (Robert et al., 2014; Sauls et al., 2016; Willis and Huang, 2017). The adder model supposes that all cells add a constant growth increment from birth to division regardless of birth size and thus gives a slope of 0 (Taheri-Araghi et al., 2015a). The adder-per-origin model posits that all cells grow a constant length from initiation to initiation, and then divide a constant time after initiation. However, a constant adder from initiation to initiation mathematically reduces to an adder correlation from birth to division, giving a slope of zero (Amir, 2014). Light blue bars represent *l*_b_ vs. Δ*l*_bd_ slope measurements from populations of model bacteria. *E. coli* and *B. subtilis* from Taheri-Araghi et al. (2015a), *C. crescentus* from Campos et al. (2014), *P. aeruginosa* from Deforet et al. (2015). Dark blue bars represent *l*_b_ vs. Δ*l*_bd_ slope measurements from mycobacterial populations. *M. smegmatis* rich media, carbon limited, and BCG from Logsdon et al. (2017). *M. smegmatis* acetate and pyruvate from Priestman et al. (2017). ^∗^The timer model was shown in several studies to be incompatible with generation of stable bacterial cell size homeostasis (Trucco and Bell, 1970; Robert et al., 2014).

Historically, models of cell size control used measurements of size or time to relate events within the cell cycle of exponentially growing cells (“timer” or “sizer” models) (**Figures 3A,B**) (Koch and Schaechter, 1962; Robert et al., 2014; Willis and Huang, 2017). Time increments measure the minutes or hours between events, (e.g., cells grow 100 min before division), while size measurements record the absolute length at an event (e.g., cells divide once reaching 7 μm in length) (**Figures 3A,B**). Models of cell size control used one or both types of measurements simultaneously to describe bacterial cell division. Model bacteria were largely considered to follow a sizer model as timer models are very sensitive to slight differences in exponential growth rates among cells and thus do not reach balanced growth and size homeostasis (Trucco and Bell, 1970; Robert et al., 2014). A key sizer model prediction is that birth size plotted against birth to division elongation is negatively correlated, because smaller cells must grow more to reach the absolute cell size for division (**Figure 3E**) (Koch and Schaechter, 1962; Sauls et al., 2016). Alternatively, a non-correlation or negative correlation between these two parameters had been interpreted as a time-based mechanism (**Figure 3E**) (Sveiczer et al., 1996; Robert et al., 2014; Willis and Huang, 2017). Therefore, when we first observed no correlation between *M. smegmatis* birth size and elongation, we concluded that mycobacteria use a time-based mechanism to control cell division (Aldridge et al., 2012).

Soon after, a new framework for understanding cell size control was introduced centered on the amount of growth rather than absolute size or elapsed time. Growth increments measure the change in length between two events (e.g., cells divide after adding 4 μm to their original length). The new model is referred to as an incremental or adder model of cell size control and postulates that every cell grows a constant length between birth and division, regardless of birth size (**Figure 3C**) (Campos et al., 2014; Taheri-Araghi et al., 2015a). With this in mind, a non-correlation between birth size and birth to division elongation (as seen in *M. smegmatis*) could be reinterpreted as arising from an adder (**Figure 3E**). Several studies adapted this model to predict that a constant change in cell length (Δ*l*_bd_), surface area, or volume from birth to division regulates cell size (Amir, 2014; Campos et al., 2014; Deforet et al., 2015; Taheri-Araghi et al., 2015a; Harris and Theriot, 2016; Sauls et al., 2016; Soifer et al., 2016). In all cases, homeostasis is achieved because average birth size converges to the length of the increment added at each generation (Jun and Taheri-Araghi, 2015). Adding a constant length increment compensates for aberrant birth sizes by correcting outlier cells back to the population mean with exponential decay at each round of division (Taheri-Araghi et al., 2015a).

Thus, until recently, it was thought that mycobacteria used an adder mechanism of cell size control. Data from several labs corroborated the constant growth increment from birth to division in populations of *M. smegmatis* in rich medium (**Figure 3E**) (Aldridge et al., 2012; Santi et al., 2013; Logsdon et al., 2017; Priestman et al., 2017). Some of these data were produced several years before the mathematical framework for the adder model of cell size control was introduced, and have been retrospectively interpreted through this lens (Aldridge et al., 2012; Santi et al., 2013; Sauls et al., 2016). However, as recent work has shown, this adder behavior is not consistent across growth conditions (**Figure 3E**).

Several studies tested the growth and division of mycobacteria in sub-optimal carbon conditions because intracellular infection is thought to be carbon limited, with cholesterol serving as a key carbon source for *M. tuberculosis* during persistent infection (Pandey and Sassetti, 2008). Cell growth measurements of *M. smegmatis* cells grown with carbon restricted medium or with the cholesterol by-products acetate or pyruvate as the primary carbon source significantly deviated from expected adder correlations (**Figure 3E**). When cells are grown in acetate as a primary carbon source, accelerators continue to follow the adder principle but alternators are not consistent with an adder, showing a significantly negative correlation between birth length and growth birth to division (**Figure 3E**) (Priestman et al., 2017). Both accelerator and alternator cell populations grown in pyruvate and carbon limited medium deviate from the adder model and show a significantly negative correlation between birth length and growth birth to division (**Figure 3E**) (Logsdon et al., 2017; Priestman et al., 2017). BCG cells growing in rich medium also deviate from the adder model expectation (**Figure 3E**) (Logsdon et al., 2017). In other bacteria that follow the adder model, the constant growth correlation is maintained across many nutrient rich or poor growth media. Instead of shifting the correlation between birth length and total elongation, species like *E. coli* and *B. subtilis* change the value of the length added to cell size to allow cell sizes to adapt to nutrient poor or rich media (Taheri-Araghi et al., 2015a). Therefore, the negative correlation between birth length and birth to division growth indicates that the adder model cannot capture size control in the slow growing mycobacterium species BCG or in *M. smegmatis* growing in nutrient poor medium (**Figure 3E**). It is quite remarkable that the model mycobacterium *M. smegmatis* appeared to be an adder in standard, rich-growth conditions. This apparent (but not true) adder behavior also emphasizes the importance of mathematical modeling and nutritional perturbations in studying bacterial cell size control.

Because the correlation between birth size and growth shifts to become negative in nutrient limited growth conditions (Logsdon et al., 2017; Priestman et al., 2017), this *M. smegmatis* birth to division adder correlation in rich medium presents as an emergent property rather than the actual mechanism of size control (**Figure 3E**). To look beyond simple birth to division models of size control, many studies evaluate the relationship between cell size and intermediate cell cycle events (Cooper and Helmstetter, 1968; Donachie, 1968; Hill et al., 2012; Adiciptaningrum et al., 2015; Ho and Amir, 2015; Soifer et al., 2016; Wallden et al., 2016; Zheng et al., 2016; Banerjee et al., 2017; Osella et al., 2017). Several studies investigating the quantitative basis of cell cycle and size control showed that an initiation-to-initiation adder correlation mathematically reduces to an adder correlation from birth to division (**Figure 3E**) (Amir, 2014; Ho and Amir, 2015). On this basis, it was proposed that cell size control in bacteria is simply a consequence of a mechanism evolved to control the number of replication forks in bacteria (Amir, 2017). These observations served as the backbone of the adder-per-origin model, in which cells add a constant increment from initiation to initiation and then divide a constant time after initiation (**Figure 3D**). The adder-per-origin model has the appealing property of regulating cell size based on the growth rate and number of origins in the cell (particularly in bacteria that use multifork replication). Ori number must have an exponential dependence on growth rate, to ensure that cell growth does not outpace DNA replication (Schaechter et al., 1958; Ho and Amir, 2015). Therefore, an initiation dependent model could regulate mycobacterial cells with different growth rates (e.g., accelerator and alternator sister cells) and cells with multiple initiations per cell cycle (e.g., cells with an E period or multifork replication).

**FIGURE 4:**
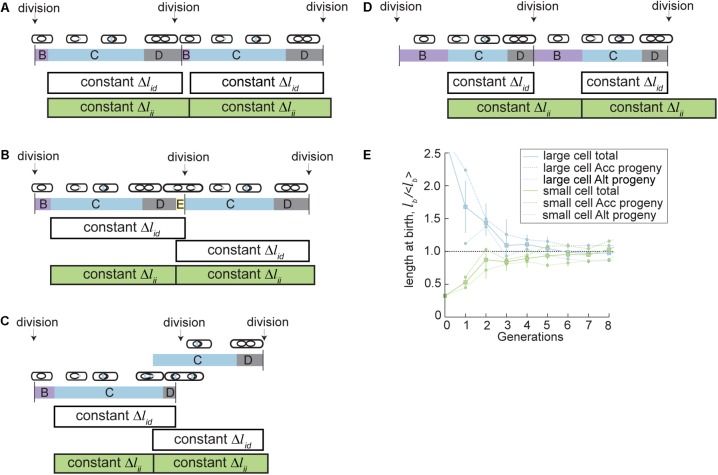
A parallel adder model describes mycobacterial cell size control in fast and slow growth conditions. In the parallel adder model of cell size control, cells grow a constant length between two initiations (Δ*l*_ii_, green bars), and simultaneously grow a constant length between initiation and division (Δ*l*_id_, white bars). The mean of these two growth increments represents the average birth size. Initiation of DNA replication occurs at the beginning of C period (blue). **(A)** Parallel adder growth increments (Δ*l*_id_ and Δ*l*_ii_) describe *M. smegmatis* cell cycle and division dynamics during fast growth. **(B)** The similar length of *M. smegmatis* parallel adder growth increments (Δ*l*_id_ and Δ*l*_ii_) in fast growth conditions can cause stochastic overlaps in initiation and division, leading to reinitiation during D period, called E period (shown in yellow). The parallel adder accommodates stochastic re-initiation events including E period and allows recovery of population cell cycle dynamics. **(C)** The similar length of *M. smegmatis* parallel adder growth increments (Δ*l*_id_ and Δ*l*_ii_) in fast growth conditions can cause stochastic overlaps in initiation and division, leading to reinitiation during C period, called multifork replication (shown by overlapping C periods). The parallel adder accommodates stochastic re-initiation events and allows recovery of population cell cycle dynamics. **(D)** The parallel adder model shifts relative lengths of Δ*l*_id_ and Δ*l*_ii_ growth increments during slow growth conditions (carbon limited *M. smegmatis* or BCG in rich medium) to accommodate differences in cell size, growth rate, and cell cycle dynamics during slow growth. However, constant Δ*l*_id_ and Δ*l*_ii_ increments pattern is maintained. **(E)** The parallel adder model leads to cell size convergence in accelerator and alternator subpopulations, despite innate asymmetry. Plot showing parallel adder simulations for progeny of hypothetical cells born extremely large (blue) or small (green). The average cell birth length with SEM bars over eight generations is plotted for a hypothetical cell (accelerator) born 2.53x the population average and a hypothetical cell (alternator) born 0.33x the population average. Average sizes of accelerator and alternator cell progeny from each hypothetical progenitor cell are also plotted with SEM bars over seven generations. Figure **(E)** is adapted from Logsdon et al. (2017).

We recently developed an initiation-dependent model of cell size control that describes the specific growth and division properties in mycobacteria. These properties include asymmetric polar growth and division, shifting lengths of B and E period during slow growth, and increased variability in cell size during slow growth (Logsdon et al., 2017). The new model is named the parallel adder model because it is based on a regulatory mechanism in which cells add two constant growth increments simultaneously. One growth increment spans between two DNA replication initiation events (Δ*l*_ii_) while the other increment spans initiation to division (Δ*l*_id_) (**Figure 4A**). Together, these parallel increments allow coupled regulation of cell division and cell cycle dynamics. When directly tested against previously established models of cell size control, including adder and adder-per-origin models, the parallel adder model was significantly better fit to 13 key cell cycle and cell size measurements in *M. smegmatis* and BCG (Logsdon et al., 2017). The primary difference between initiation-to-initiation adder models in mycobacteria (parallel adder) versus previously studied *E. coli* (adder-per-origin) is that *E. coli* cell division occurs a constant time after initiation, while mycobacteria divide after a constant growth increment from initiation to division (Amir, 2014; Ho and Amir, 2015; Logsdon et al., 2017). However, in both cases initiation of DNA replication provides the key checkpoint controlling cell division and initiation in the next generation.

Importantly, the increments of the parallel adder model shift to allow cells to optimize total size in a variety of growth environments (Logsdon et al., 2017). Parallel adder increments can adjust in total length and in relation to one another, allowing shifts in cell cycle timing and variability, most notably in B period and E period. In rich growth conditions, the Δ*l*_ii_ and Δ*l*_id_ growth increments are similar in length (Logsdon et al., 2017). This necessitates a shorter pre-initiation period (B period) and can often cause an overlap of initiation periods before division, meaning that the mother cell experiences E period (**Figure 4B**) or multifork replication (**Figure 4C**) and daughter cells entirely skip B. However, in BCG and carbon limited *M. smegmatis* cells, Δ*l*_ii_ increments are much longer than Δ*l*_id_, leading to longer B periods and lowered frequency of both E period and multifork replication (**Figure 4D**). In this way, cell cycles are adjusted to create increased variability in timing under nutrient stress or in slow growing mycobacteria.

With a parallel adder, size convergence occurs by the same principle as other adder models, e.g., if cells add a constant length regardless of their initial size, cells smaller than average will increase in size and cells larger than average will decrease in size until they reach the population average (which is equal to the size of the growth increment added) over several generations (**Figure 4E**) (Amir, 2014; Taheri-Araghi et al., 2015a). The parallel adder regulates birth size by setting the cell length at birth equal to the average of the Δ*l*_ii_ and Δ*l*_id_ increments. Δ*l*_ii_ and Δ*l*_id_ measurements are specific for accelerator and alternator subtypes, and so accelerator and alternator cells converge to distinct average cell sizes (Logsdon et al., 2017). This creates increased variability in mycobacterial size compared to other studied bacteria, and reflects the distinct size, growth, and adder increment properties measured in accelerator vs. alternator cells. As an example, an accelerator cell will add an accelerator specific Δ*l*_ii_ increment as well as an accelerator specific Δ*l*_id_ increment. Averaging the lengths of those two growth increments allows calculation of the birth size of the average accelerator progeny. Accelerator specific increments are larger than alternator specific increments, causing the average accelerator cell size to be larger than that of the larger alternator cell size (**Figure 4E**). Another well characterized asymmetrically dividing bacterial species, *C. crescentus*, exhibits an asymmetric incremental growth pattern that is opposite that of *M. smegmatis. C. crescentus* large (stalked) cells add a smaller increment and small (swarmer) cells add a larger increment to compensate for asymmetric birth sizes, leading to less variable birth and division sizes (Campos et al., 2014). Though *M. smegmatis* does not have this mechanism of compensating for asymmetric birth sizes, both accelerator and alternator cell types can recover from aberrant large or small birth sizes to reach homeostasis with the parallel adder model (**Figure 4E**). Therefore, the total population average can also reach homeostasis, albeit slightly more variable than most bacteria (Logsdon et al., 2017).

In the parallel adder model, initiation of DNA replication is the key checkpoint regulating cell division and size control under many conditions. Additional support for an initiation dependent model comes from a recent study identifying a novel essential component of the mycobacterial replication machinery called Rv0004. Rv0004 interacts with helicase loader DnaB and affects the interaction of DnaA and DnaB (Mann et al., 2017). When Rv0004 is depleted from cells growing in rich medium, variability in *M. smegmatis* birth size more than doubles, from a CV of 18% to a CV of 40% (Mann et al., 2017). This increased variability indicates that the process of initiation is molecularly coupled to division, as interrupting replisome assembly disrupts division and cell size regulation. It is possible though that there are additional “backup” mechanisms regulating cell division in the absence of initiation, as many of these cells still do divide, albeit with less size consistency than WT cells (Mann et al., 2017). Additional genetic or chemical inhibition of cell cycle processes will allow us to tease apart regulatory mechanisms of cell division and size control.

There are many additional and important questions regarding size control in mycobacteria to be addressed with future research. While the parallel adder describes mycobacterial growth under all conditions studied thus far (*M. smegmatis* rich growth, *M. smegmatis* carbon limitation, and BCG rich growth), it is possible that other growth conditions may reveal inconsistencies with the model and require further assessment or model modification. Additionally, it has yet to be determined whether any of the developed models of cell size control apply to *M. tuberculosis*. Cell size control models could provide an important tool for probing *M. tuberculosis* population dynamics during infection. Though correlations between cell size and antibiotic tolerance hold under controlled growth conditions, it is not known if they apply to variable and stressful conditions present in a host (Lin et al., 2014; Cadena et al., 2017; Martin et al., 2017). Cell size and cell cycle dynamics are crucial to understand during infection because of previously observed correlations with stress tolerance (Aldridge et al., 2012; Manina et al., 2015; Richardson et al., 2016; Vijay et al., 2017).

In summary, mycobacteria actively create population heterogeneity through growth, division, and cell cycle patterns. The extent of variability is partially determined by growth conditions and environmental stress experienced by mycobacterial cells. Differences in cell size within a population of mycobacteria increase under a wide variety of stressors. *M. smegmatis* and BCG coordinate basic cell cycle properties with their increased variation under stress using a unique model of cell size control. The parallel adder model accommodates inherent variation in cell cycle and growth that is likely important for population survival. Models of the cell cycle and size control could be particularly useful in predicting bacterial population dynamics during infection leading to the development of persistent subpopulations. Understanding the increase in cell-to-cell variability observed in *M. tuberculosis* cells under stress could aid in identifying and targeting the molecular pathways responsible for population heterogeneity and stress tolerance.

Despite the irregularities of mycobacteria compared to other well-studied rod shaped organisms, mycobacterial studies may provide a means of untangling many unanswered questions about bacterial physiology. Mycobacteria actively create exaggerated heterogeneity among closely related cells, allowing the resolution of bacterial population structure with a level of detail never before observed. We can assess functional differences created between (presumably) genetically identical sister cells, and easily identify phenotypic characteristics that allow survival under stress. Mycobacteria do not follow commonly observed patterns of growth, division, and size control, forcing researchers to look beyond established mechanisms developed with data from other bacteria. It became necessary to develop a novel model to account for innate asymmetry and pre-determined cell-to-cell variation observed in mycobacteria. The development of models to describe asymmetric mycobacterial cell properties may provide frameworks through which to understand other asymmetric or pole growing bacteria. In fact, when considering the diversity of bacterial cell sizes and shapes, ranging from filaments to spirochetes to cocci, and spanning sizes of 0.3–750 μm, we cannot help but wonder if mycobacteria are not so unusual after all.

## Author Contributions

ML and BA conceived of the review topic and structure, wrote the manuscript, and edited the manuscript.

## Conflict of Interest Statement

The authors declare that the research was conducted in the absence of any commercial or financial relationships that could be construed as a potential conflict of interest.
